# Recovering Spatially-Varying Cell-Specific Gene Co-expression Networks for Single-Cell Spatial Expression Data

**DOI:** 10.3389/fgene.2021.656637

**Published:** 2021-04-26

**Authors:** Jinge Yu, Xiangyu Luo

**Affiliations:** Institute of Statistics and Big Data, Renmin University of China, Beijing, China

**Keywords:** Bayesian posterior estimates, cell-specific, gene co-expression network, prediction, single-cell spatial expression, neighborhood

## Abstract

Recent advances in single-cell technologies enable spatial expression profiling at the cell level, making it possible to elucidate spatial changes of cell-specific genomic features. The gene co-expression network is an important feature that encodes the gene-gene marginal dependence structure and allows for the functional annotation of highly connected genes. In this paper, we design a simple and computationally efficient two-step algorithm to recover spatially-varying cell-specific gene co-expression networks for single-cell spatial expression data. The algorithm first estimates the gene expression covariance matrix for each cell type and then leverages the spatial locations of cells to construct cell-specific networks. The second step uses expression covariance matrices estimated in step one and label information from neighboring cells as an empirical prior to obtain thresholded Bayesian posterior estimates. After completing estimates for each cell, this algorithm can further predict or interpolate gene co-expression networks on tissue positions where cells are not captured. In the simulation study, the comparison against the traditional cell-type-specific network algorithms and the cell-specific network method but without incorporating spatial information highlights the advantages of the proposed algorithm in estimation accuracy. We also applied our algorithm to real-world datasets and found some meaningful biological results. The accompanied software is available on https://github.com/jingeyu/CSSN.

## 1. Introduction

The last decade witnesses that the single-cell RNA-sequencing has revolutionized the focus of genomic analyses from bulk samples to single cells, but the technology loses important cell spatial information during tissue dissociation. Fortunately, recent technological advances have allowed for measurements of the gene expression levels at single-cell resolution while retaining the coordinates of cells in the tissue section (Chen et al., 2015; Moffitt et al., 2018; Wang et al., 2018). Specifically, various spatially resolved transcriptomic techniques have been developed to profile single-cell expression with cells' spatial information, including MERFISH (Chen et al., 2015), seqFISH (Lubeck et al., 2014), and FISSEQ (Lee et al., 2014), just to name a few. They are mainly based on either *in situ* hybridization or *in situ* sequencing. Fluorescence *in situ* hybridization (FISH) based approaches can measure hundreds of preselected marker genes, while *in situ* sequencing based approaches can measure thousands of transcripts. Moreover, different techniques may have different strategies to capture transcriptomic spatial information. For example, MERFISH adopts an imaging-based way to map transcriptomic spatial organization for a three-dimensional tissue region. Usually, the region needs to be first sectioned into evenly spaced slices, and MERFISH is then performed on these slices, resulting in two-dimensional localization information. The information makes it possible to investigate spatial and functional organization of cells.

The amazing biological progress also offers rich opportunities to investigate the spatial patterns of cell-specific genomic features (Zhang et al., 2020). When features are genes, Sun et al. (2020) developed a statistical method to identify genes with spatially differential expressions. Li D. et al. (2020) utilized an expert system to predict signaling gene expression using information from nearby cells. However, as observed gene expressions may suffer from systematic biases (Köster et al., 2019) and are dynamically driven by an underlying regulation system, it is of more interest to study a more stable feature—gene co-expression network—(Dai et al., 2019) and learn its spatial pattern from one cell to another.

The gene co-expression network (Butte and Kohane, 2000; Stuart et al., 2003; Carter et al., 2004) can be encoded in an undirected graph, where nodes correspond to genes and an edge between nodes A and B indicates a significant association between expressions of the genes A and B. It has important biological applications including functional annotation for a set of unknown but highly connected genes (Serin et al., 2016) and single cell expression simulation (Tian et al., 2021). The pipeline to construct gene co-expression networks usually consists of two steps (Zhang and Horvath, 2005). In step one, we adopt a similarity measure (e.g., the absolute value of Pearson correlation) and calculate the similarity for all pairs of genes. In step two, we choose a threshold and genes with similarity larger than the threshold are thought of as co-expressed. Following the pipeline, Dai et al. (2019) proposed a hypothesis testing based approach to estimate cell-specific gene co-expression network, which is a breakthrough from “cell-type-specific” to “cell-specific” since most computational network methods for single-cell expression are restricted to a group of cells and ignore cell heterogeneity. Li L. et al. (2020) extends the approach to a conditional cell-specific network situation. Unfortunately, the method (Dai et al., 2019) does not incorporate the spatial information of cells and thus may lose power in estimating cell-specific gene co-expression structures, let alone carry out network prediction given a new cell location in the tissue.

**Figure 1:**
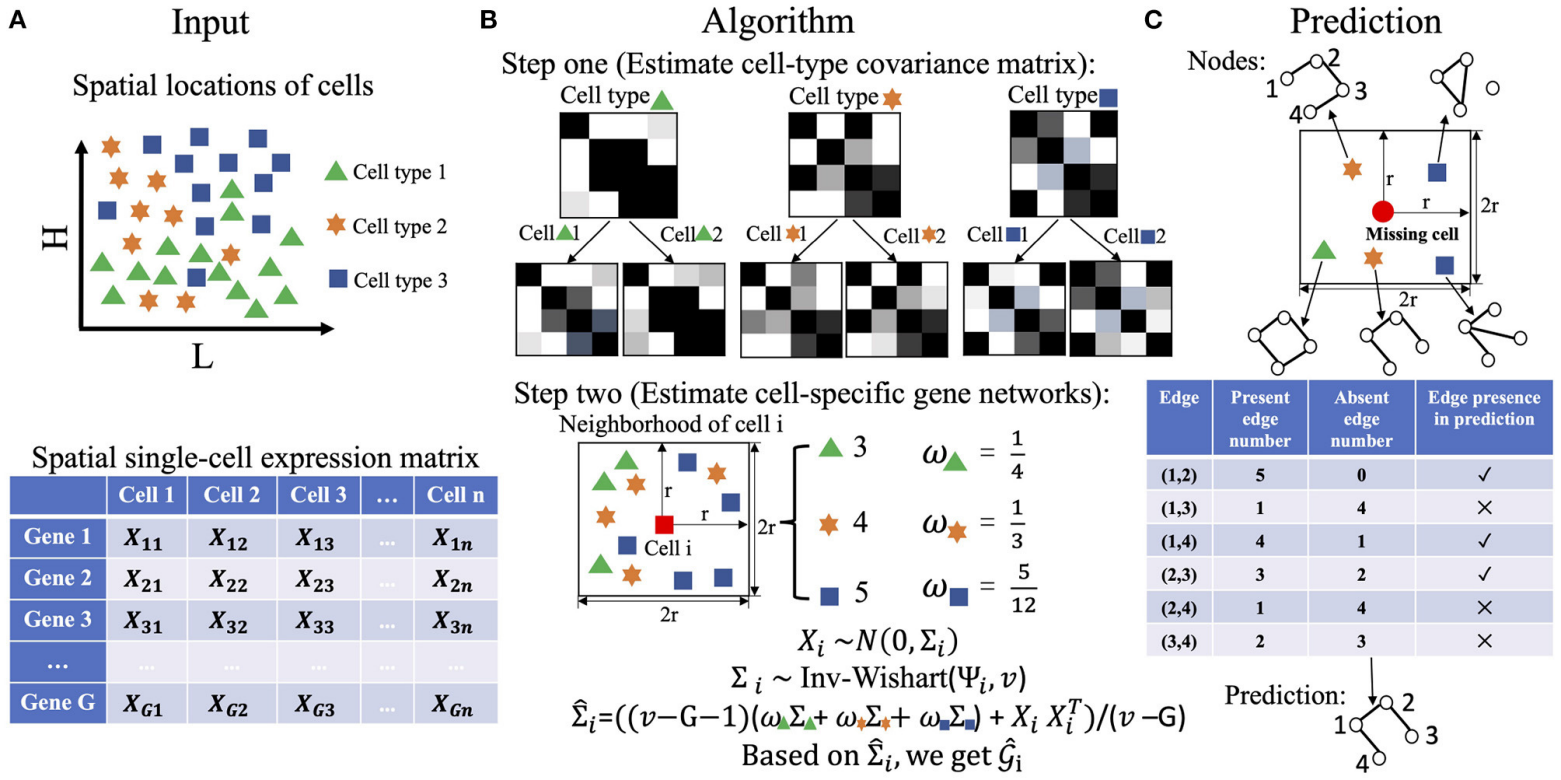
An illustration of the two-step algorithm. **(A)** The input of the algorithm, including spatial coordinates of cells, the gene-cell expression matrix, and cell class information. Different shapes and colors represent different types of cells. **(B)** The two steps in the proposed algorithm. We first estimate cell-type covariance matrices, and then we use those estimates to refine cell-specific gene co-expression networks. **(C)** Gene co-expression network prediction based on the estimates from **(B)**.

To overcome the challenges, we present an easy-to-implement and computationally efficient two-step algorithm to recover cell-specific gene co-expression networks for single-cell spatial expression data. The input of the proposed algorithm is comprised of the spatial locations of cells, cell labels, as well as the gene-cell expression matrix (Figure 1A). If cell label information is not available, we can first carry out clustering using single-cell expression data clustering tools (Butler et al., 2018; Stuart et al., 2019). In step one, we estimate the sample expression covariance matrix for each cell type, which serves as the “average” of the cell-specific covariance matrices in a given cell type (Figure 1B). In step two, for any given cell, we find its appropriate neighborhood and combine the cell label proportions in the neighborhood and the cell-type covariance matrices estimated in step one to assign an empirical prior to the covariance matrix of that cell. Subsequently, we apply the Bayes' rule to obtain the posterior mean estimates, transform it to the correlation matrix, and select a threshold to shrink absolute values of correlations less than it to zero, resulting in the cell's gene co-expression network (Figure 1B). After completing the estimates for each cell, we can further predict the network structures for a position where cells are not detected. We set a neighborhood of the location like in the estimation step two, and then an edge is present if and only if this edge appears more than or equal to half times among the gene networks of its neighboring cells (Figure 1C).

In the following, we introduce our proposed algorithm in detail in section 2. Section 3 provides the simulation study to compare the two-step algorithm against competing methods including traditional network construction methods (Zhang and Horvath, 2005) based on a group of cells and the cell-specific network construction approach (Dai et al., 2019). We use MERFISH data to demonstrate the good utility of the algorithm in section 4 and conclude the paper with a discussion in section 5.

## 2. Method

We first give some notations to clearly express the data preprocessing and our algorithm. Suppose that expression levels of *G* genes in *n* cells are measured and the expression of gene *g* in cell *i* is denoted by *X*_*gi*_. We let **X** = (_*X*_*gi*_)*G*×*n*_ represent the gene-cell expression matrix and use **X**_*i*_ to denote the *ith* column vector. The coordinates of cell *i* in the tissue section are denoted by (ℓ_*i*_, *h*_*i*_). We further assume that cells are from *K* distinct cell types and *C*_*i*_ indicates the membership of cell *i*. In other words, *C*_*i*_ = *k* (*k* = 1, …, *K*) implies that cell *i* belongs to cell type *k*. Notice that the cell labels **C** = (*C*_1_, …, *C*_*n*_) are assumed to be known in advance, and in case the cell label information is not available we can cluster cells using off-the-shelf single-cell expression tools. *n*_*k*_ is the cell number in cell type *k*, and **S**_*k*_ represents the index set {*i*:*C*_*i*_ = *k*}.

During data preprocessing, we need to normalize raw read count data to reduce the effects of different library sizes and other systematic biases. As we are interested in the pairwise gene correlations, the normalized expression values are further centered to zero and scaled to variance one within each cell type. If we still use *X*_*gi*_ to represent the normalized expression, then the transformed value is as follows. When *C*_*i*_ = *k*,

$$\begin{array}{r} {\tilde{X}}_{gi}=\frac{X_{gi}-\frac{1}{n_{k}}\sum_{j\in {\text{S}}_{k}}X_{gj}}{\sqrt{\frac{1}{n_{k}-1}\sum_{j\in {\text{S}}_{k}}{\left(X_{gj}-\frac{1}{n_{k}}\sum_{j\in S_{k}}X_{gj}\right)}^{2}}}. \end{array}$$

Next, we utilize the scaled expression matrix $\tilde{\text{X}}={\left({\tilde{X}}_{gi}\right)}_{G\times n}$ and its *ith* column vector ${\tilde{\text{X}}}_{i}$ in our algorithm.

In step one, we derive the sample expression covariance matrix for each cell type, which serves as the “average” of all cell-specific expression covariance matrices in that cell type and hence can be treated as an initial and coarse-grained estimate of the expression covariance matrix for each cell. Specifically, for cell type *k*, its sample expression covariance matrix is estimated by ${\hat{\Sigma }}^{\left(k\right)}:=\frac{1}{n_{k}-1}\underset{i\in {\text{S}}_{k}}{\sum }{\tilde{\text{X}}}_{i}{\tilde{\text{X}}}_{i}^{T}$ (1 ≤ *k* ≤ *K*).

In step two, suppose the gene expression covariance matrix of cell *i* is denoted by **Σ**_*i*_. Biologically, **Σ**_*i*_ depends on both cell *i*'s cell type as well as cell *i*'s spatial circumstances. Taking this into account, we assume the following Bayesian statistical model for the observations,

(1) $$\begin{array}{rc} {\tilde{\text{X}}}_{i} & \sim N\left(0,\Sigma_{i}\right) \end{array}$$

(2) $$\begin{array}{rc} \Sigma_{i} & \sim W^{-1}\left(\Psi_{i},\nu \right), \end{array}$$

where $N\left(0,\Sigma_{i}\right)$ is a multivariate normal distribution with mean vector zero and covariance matrix Σ_*i*_, and $W^{-1}\left(\Psi_{i},\nu \right)$ is an inverse-Wishart distribution with scale matrix **Ψ**_*i*_ and ν degrees of freedom.

Equation (1) corresponds to the data-generating mechanism in which cell *i*'s observation is sampled from its own distribution parameterized by **Σ**_*i*_. In the normal distribution, a zero element in **Σ**_*i*_ indicates that the corresponding two genes are independent, so **Σ**_*i*_ fully captures the gene co-expression network structure of cell *i*. Equation (2) reflects that we need to provide prior information for **Σ**_*i*_ to stabilize the estimate of **Σ**_*i*_; otherwise, only one sample is available, making the common maximal likelihood estimate very sensitive. We employ the inverse-Wishart distribution here as it is conjugate to the multivariate normal distribution (Gelman et al., 2013), which can enhance fast calculation of posterior estimates. Accordingly, we aim to borrow information from cell *i*'s neighbors to define the hyper-parameter in the prior—the scale matrix **Ψ**_*i*_.

For each cell, we define its neighborhood as a square region with side length 2*r* and center at the location of the cell (Figure 1B). The choice of *r* depends on the cell density in the tissue section and our knowledge about the number of informative neighboring cells. We define the cell density as the ratio of the cell number (*n*) to the area where cells locate (*A*). As the area shape is often like a rectangle, we estimate *A* by $Â:=\left(\underset{i}{\max}\ell_{i}-\underset{i}{\min}\ell_{i}\right)\left(\underset{i}{\max}h_{i}-\underset{i}{\min}h_{i}\right)$. If we believe that on average each cell has *m*_*info*_ informative neighboring cells, we then have the relationship $n/Â\times 4r^{2}=m_{info}$, leading to $r=0.5\sqrt{m_{info}Â/n}$. Based on our experience, we set *m*_*info*_ = 70 throughout our paper. Subsequently, we count the number of cells in this square region for each cell type and calculate proportions (ω_*i*1_, …, ω_*iK*_) with ω_*ik*_≥0 and $\overset{K}{\underset{k=1}{\sum }}\omega_{ik}=1$, where ω_*ik*_ is the proportion of type *k* cells in the neighborhood of cell *i*.

Next, we assign the weighted value $\overset{K}{\underset{k=1}{\sum }}\omega_{ik}{\hat{\Sigma }}^{\left(k\right)}$ to the prior mean of **Σ**_*i*_, which is **Ψ**_*i*_/(ν−*G*−1), resulting in the scale matrix $\Psi_{i}=\left(\nu -G-1\right)\overset{K}{\underset{k=1}{\sum }}\omega_{ik}{\hat{\Sigma }}^{\left(k\right)}$. This prior reflects the information of nearby cells and helps stabilize the estimate of **Σ**_*i*_. We remark that the choice of the hyper-parameter **Ψ**_*i*_ depends on the data we are analyzing, so strictly speaking the approach is not fully Bayesian (Gelman et al., 2013).

Given the assigned prior, we estimate **Σ**_*i*_ by the posterior mean,

$$\begin{array}{r} {\hat{\Sigma }}_{i}:=E\left(\Sigma_{i}|{\tilde{\text{X}}}_{i}\right)=\frac{1}{\nu -G}\left(\Psi_{i}+{\tilde{\text{X}}}_{i}{\tilde{\text{X}}}_{i}^{T}\right)\\ \;=\frac{1}{\nu -G}\left(\left(\nu -G-1\right)\overset{K}{\underset{k=1}{\sum }}\omega_{ik}{\hat{\Sigma }}^{\left(k\right)}+{\tilde{\text{X}}}_{i}{\tilde{\text{X}}}_{i}^{T}\right), \end{array}$$

where we set ν to 2*G* depending on the number of genes. ${\hat{\Sigma }}_{i}$ is then transformed to its corresponding correlation matrix ${\hat{\text{R}}}_{i}=\text{diag}{\left({\hat{\Sigma }}_{i}\right)}^{-1/2}{\hat{\Sigma }}_{i}\text{diag}{\left({\hat{\Sigma }}_{i}\right)}^{-1/2}$, where $\text{diag}\left({\hat{\Sigma }}_{i}\right)$ is a diagonal matrix with diagonal elements the same as those of ${\hat{\Sigma }}_{i}$. Finally, we select a threshold *d* (0 < *d* <1), and if the (*g*_1_, *g*_2_) element of the matrix ${\hat{\text{R}}}_{i}$, ${\hat{R}}_{i,g_{1}g_{2}}$, has an absolute value larger than *d*, then we believe there is an edge between gene *g*_1_ and *g*_2_ in the gene co-expression network of cell *i*. Algorithm 1 displays the two-step estimation procedure.


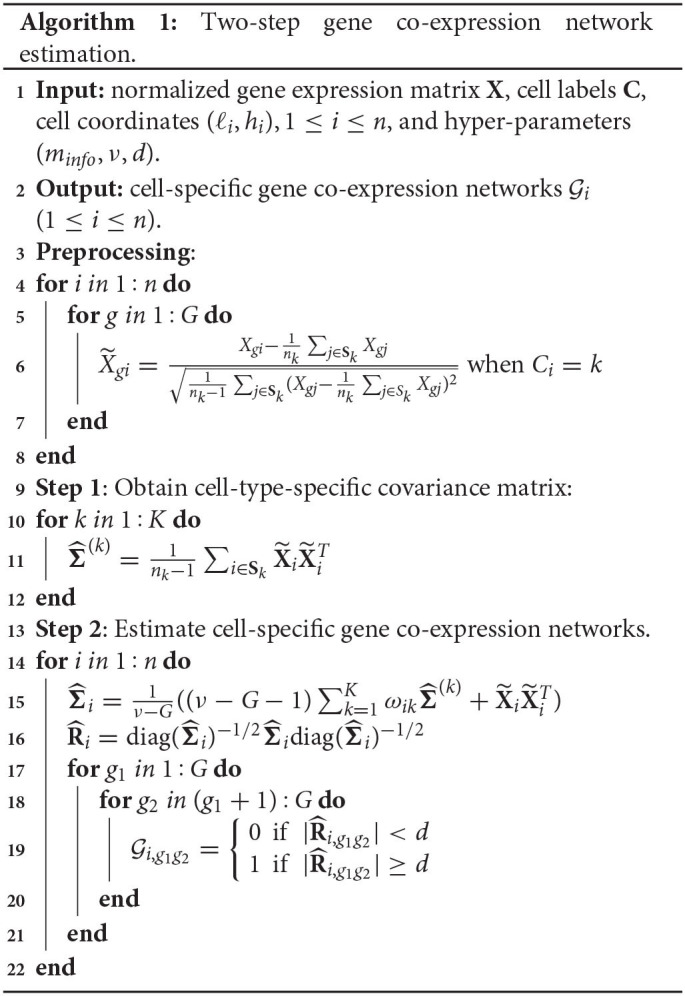


After completing the network structure estimates for all cells, we can take advantage of the estimates to predict the gene co-expression network for any missing cell with a position in the studied tissue section area. If we are interested in an undetected cell at a new location (ℓ^*^, h^*^), its gene co-expression network is constructed as follows. We first find all detected cells in the neighborhood of (ℓ^*^, h^*^), and then we believe an edge between genes *g*_1_ and *g*_2_ in the prediction if there are more connections than disconnections for this pair of genes among the gene networks of (ℓ^*^, h^*^)'s neighboring detected cells. Algorithm 2 shows the steps of making gene co-expression network predictions.


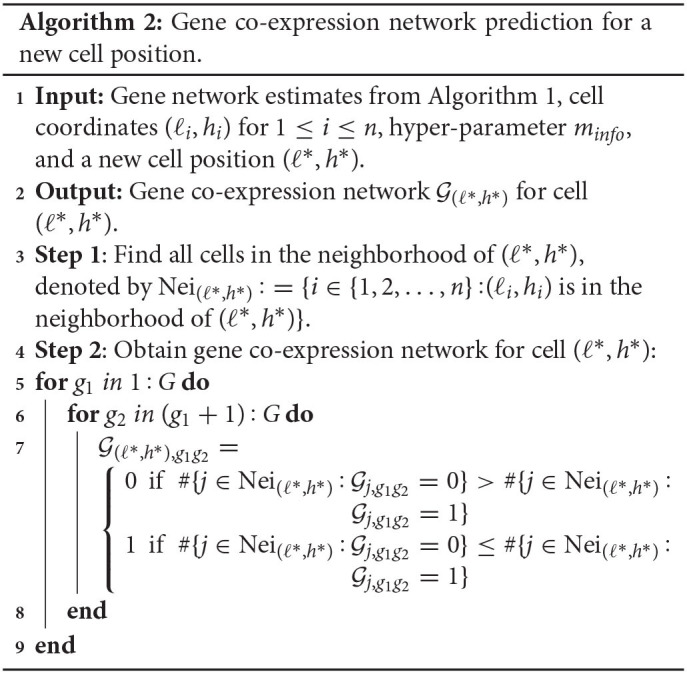


## 3. Simulation Study

**Figure 2:**
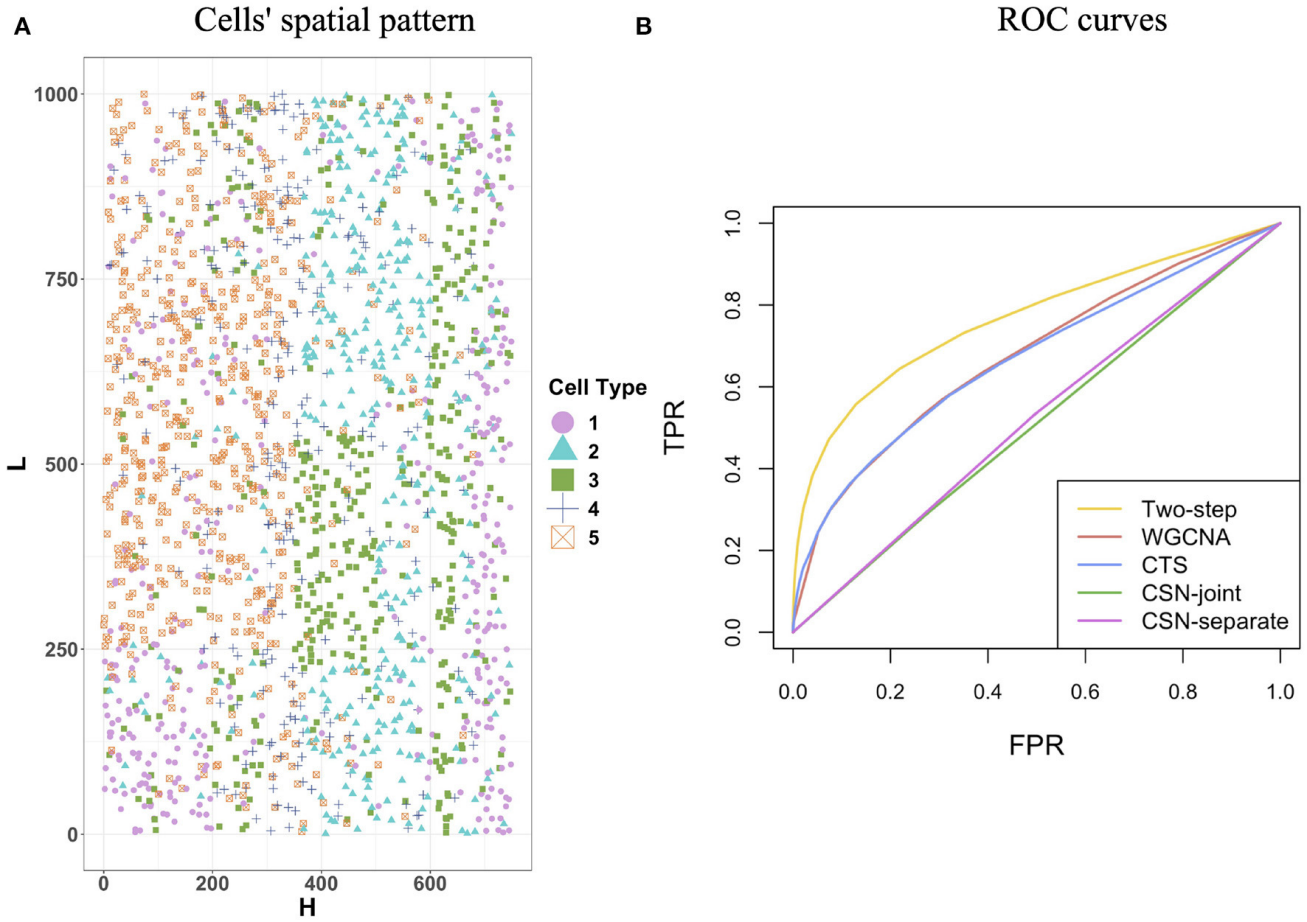
Cells' spatial pattern and performance comparisons on the network structure recovery. **(A)** Cells' spatial pattern. Different shapes and colors correspond to different types of cells. **(B)** ROC curves of the two-step algorithm, WGCNA, CTS, CSN-joint, and CSN-separate.

In this section, we used simulated data to evaluate the performance of the proposed two-step algorithm. We set the gene number *G* = 100, the cell-type number *K* = 5, and the cell number for each cell type (*n*_1_, *n*_2_, *n*_3_, *n*_4_, *n*_5_) = (394, 373, 428, 274, 529). We chose a rectangle area as the tissue section with length *L* = 1, 000 and width *H* = 750, where a total of $n=\overset{K}{\underset{k=1}{\sum }}n_{k}=1998$ cells distribute on the section and display clear spatial patterns (Figure 2A). For example, cells from cell-type 5 concentrate on the left side, while cells from cell-type 1 enrich on the right side.

We then generated cell-type-specific covariance matrices **Σ**^(*k*)^ for *k* = 1, …, *K*. Genes that work together often form a gene module, which can exhibit a block structure in the covariance matrix. Hence, the covariance matrix of each cell type was set as a block diagonal matrix, where each block was a 20 ×20 positive definite matrix. Five different modules were used for this purpose and were as follows.

- In module 1 ($M_{1}$), its (*i, j*) element $\sigma_{ij}=\rho^{\left|i-j\right|}+0.5\text{I}\left(i=j\right)$ for 1 ≤ *i* ≤ 20 and 1 ≤ *j* ≤ 20, where **I**(*A*) is an indicator function of event *A*. We took ρ = 0.7.
- In module 2 ($M_{2}$), $\sigma_{ij}={\left(1-\frac{\left|i-j\right|}{10}\right)}_{+}$, which forms a banded matrix. The function (*x*)_+_ equals *x* for *x*≥0 and zero for *x* <0.
- In module 3 ($M_{3}$), σ_*ij*_ = ρ**I**(|*i*−*j*| = 1)+1.3**I**(*i* = *j*) for ρ = −0.3.
- In module 4 ($M_{4}$), $\sigma_{j}={\left(1-\frac{\left|i-j\right|}{k}\right)}_{+}$, where *k* = ⌊*G*/2⌋.
- In module 5 ($M_{5}$), the block was *F*+ϵ*I*_20 ×20_. *I*_20 ×20_ is an identity matrix. *F* = (_*f*_*ij*_)20 ×20_ is a symmetric matrix with independent upper triangle elements *f*_*ij*_ = *unif*(−0.2, 0.8) × *Ber*(1, 0.2), where *unif*(−0.2, 0.8) is a random variable uniformly distributed on(−0.2, 0.8), and *Ber*(1, 0.2) is a Bernoulli random variable with the success probability 0.2. We set ϵ = max{−λ_min_(*F*), 0}+0.01 to ensure that *B* is positive definite, where λ_min_(*F*) is the smallest eigenvalue of *F*.

If we denote a block diagonal matrix with diagonal blocks being $M_{i_{1}}$, $M_{i_{2}}$, $M_{i_{3}}$, $M_{i_{4}}$, $M_{i_{5}}$ in the order from the upper left to the lower right by $(M_{i_{1}}$, $M_{i_{2}}$, $M_{i_{3}}$, $M_{i_{4}}$, $M_{i_{5}})$, then we specify **Σ**^(1)^=$(M_{1}$, $M_{2}$, $M_{3}$, $M_{4}$, $M_{5})$, **Σ**^(2)^=$(M_{1}$, $M_{3}$, $M_{2}$, $M_{4}$, $M_{5})$, **Σ**^(3)^=$(M_{1}$, $M_{3}$, $M_{2}$, $M_{5}$, $M_{4})$, **Σ**^(4)^=$(M_{3}$, $M_{1}$, $M_{2}$, $M_{5}$, $M_{4})$, and **Σ**^(5)^=$(M_{3}$, $M_{2}$, $M_{5}$, $M_{1}$, $M_{4})$.

Next, we generated the cell-specific gene expression covariance matrix for each cell *i*. We first obtained the neighborhood of cell *i* using *r* = 80, then calculated cell-type proportions *q*_*ik*_, 1 ≤ *k* ≤ *K* in the neighborhood, and sampled **Σ**_*i*_ from the inverse-Wishart distribution $W^{-1}\left(\overset{K}{\underset{k=1}{\sum }}q_{ik}{49\Sigma }^{\left(k\right)},G+50\right)$. Moreover, to make the network sparse and covariance matrix positive definite, non-diagonal elements in the **Σ***_i_* with absolute values less than 0.5 were shrunk to zero, and the diagonal elements in **Σ**_*i*_ were added by five. Finally, we sampled the observed gene-cell expression matrix ${\text{X}}_{i}={\left(X_{1i},\ldots ,X_{Gi}\right)}^{T}$ from the multivariate normal distribution $N\left(0,\Sigma_{i}\right)$ for 1 ≤ *i* ≤ *n*.

To show the advantage of our algorithm in estimating cell-specific gene expression matrix, we compared it against the weighted gene co-expression network analysis (denoted by WGCNA) (Zhang and Horvath, 2005), the traditional hard-thresholding cell-type-specific network estimation approach (denoted by CTS), and the cell-specific gene network estimation method that does not make use of cell spatial information (denoted by CSN, Dai et al., 2019). Specifically, in WGCNA, we first calculated pairwise gene expression similarity using the absolute values of Pearson correlations, then utilized the “soft” power adjacency function to convert the similarity matrix, and finally obtain the topological overlap matrix based on the adjacency matrix. Regarding CTS, we used the cell-type-level gene network as the estimate for each cell in that cell type. For CSN, we adopted two versions: in the joint version (CSN-joint), we used the gene-cell expression matrix for all cells as the input of the CSN method; and in the separate version (CSN-separate), we only input the gene-cell expression matrix for cells coming from one cell type, repeat the procedure for each cell type, and also obtain cell-specific network estimates. In other words, for CSN-separate, the estimations for one cell only rely on the information of cells from the same cell type.

Figure 2B provides the receiver operating characteristic (ROC) curves for network structure recovery of the proposed algorithm (denoted by two-step algorithm) and other four competing approaches (WGCNA, CTS, CSN-joint, CSN-separate). The horizontal axis represents the false positive rate (FPR), which equals the ratio of the number of edges that were wrongly detected by the method for all cells to the number of absent edges in the underlying true networks for all cells, while the vertical axis corresponds to the true positive rate (TPR), describing the ratio of the number of edges that were correctly detected by the method for all cells to the number of edges in the underlying true networks for all cells. It is observed that the ROC curve of our algorithm is uniformly over the ROC curves of the other four approaches, indicating that given any FPR the TPR of the proposed algorithm is always higher than that of the other four competing methods. As WGCNA also estimates cell-type-specific networks, it does not outperform our algorithm but is slightly better than traditional CTS.

**Figure 3:**
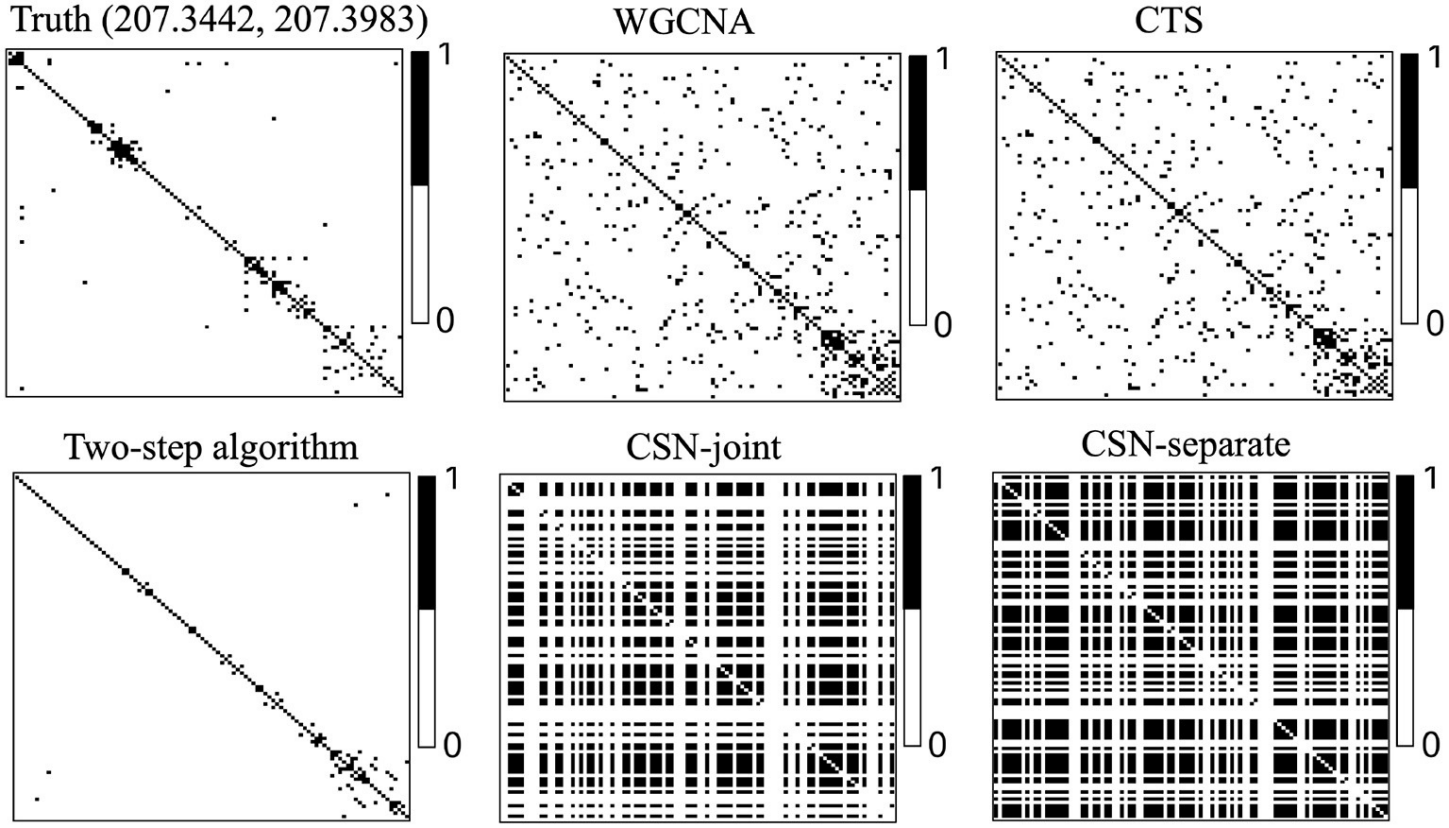
Heatmaps of estimated gene co-expression networks for different methods and underlying truth. The network heatmaps of the cell on location (207.3442, 207.3983) was used for illustration.

Figure 3 displays heatmaps of gene co-expression matrix of the cell with the coordinates (207.3442, 207.3983), both true and estimated gene co-expression matrix by two-step algorithm, WGCNA, CTS, CSN-joint, and CSN-separate are shown (the results of CSN-separate are similar to CSN-joint's). From Figure 3, we can observe that our two-step algorithm outperforms the other four methods in estimating cell-specific gene co-expression networks. To further quantify the network recovery error for these methods, we used the following error term $E:=1/n\cdot \overset{n}{\underset{i=1}{\sum }}\underset{g_{1}<g_{2}}{\sum }|G_{i,g_{1}g_{2}}-G_{i,g_{1}g_{2}}^{\text{true}}|$. For WGCNA, we chose the truncation value 0.0001 for the topological overlap matrix; for the proposed algorithm and CTS, the threshold *d* for the gene-gene correlations was chosen as 0.1; for the two CSN methods, the significance level was set at 0.01. Table 1 shows the errors based on ten replicates and indicates that the proposed method is more accurate than the others in terms of the network structure recovery.

**Table 1:** Mean errors and corresponding standard deviations of five methods.

**Methods**	**Two-step algorithm**	**WGCNA**	**CTS**	**CSN-joint**	**CSN-separate**
Mean error	288.62	484.05	484.44	1869.60	2462.09
(standard deviation)	(14.15)	(18.78)	(18.80)	(347.49)	(95.57)

**Figure 4:**
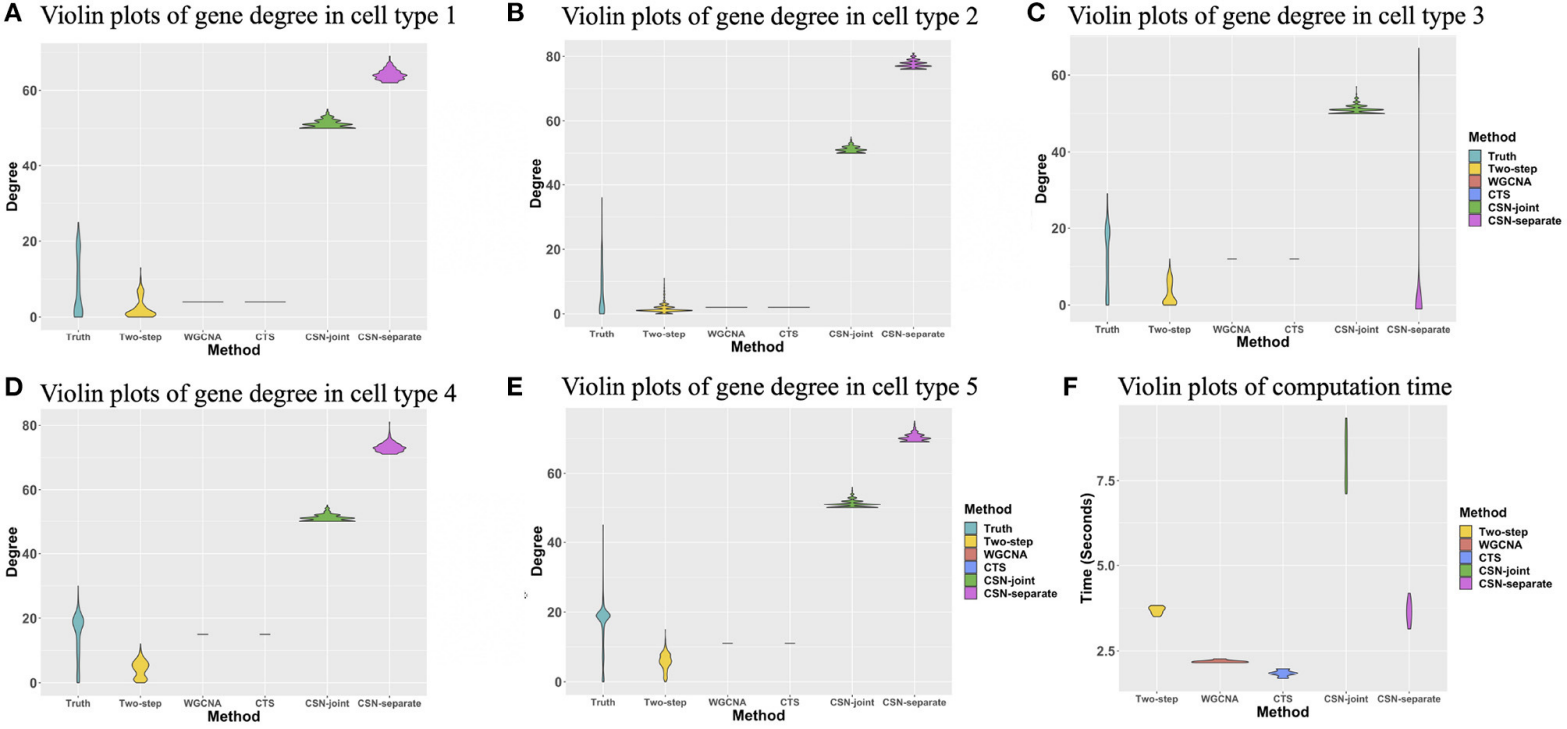
**(A–E)** Violin plots of gene degrees of gene 91 in the five cell types in the simulation study. **(F)** Violin plots of the computational time (in seconds) for the five approaches based on ten replicates.

The degree of a gene is the number of edges connected to that gene. We investigated the degree distributions of the estimated cell-specific gene co-expression network and compared it to truth and other competing approaches on one gene for each cell type. Figures 4A–E show the violin plots of the degrees of gene 91 for each cell type. We can see that the distribution created by our proposed algorithm is much closer to the underlying truth than CSN-separate and CSN-joint, while WGCNA and CTS's distributions are just horizontal line segments as their network estimates are identical for all cells in one cell type.

Figure 4F shows the violin plot for the computation time in second for these methods based on ten replicates. It is reasonable that WGCNA and CTS have the minimum computing time as they only estimate *K* cell-type-specific gene-gene network, but their performances are obviously not good. The proposed algorithm has a similar computing time to CSN-separate and is faster than CSN-joint. Hence, our algorithm not only performs well in estimating networks but also has relatively fast computing.

Given the network estimates by our method, we can easily use algorithm 2 to predict network structures for a new location. We randomly generated 50 new coordinates as the locations of 50 missing cells, simulated the true gene network of these 50 new cells following the data-generating procedure above, and then applied the prediction algorithm. The prediction error is 347.84 (in terms of *E*). WGCNA, CTS, CSN-joint, and CSN-separate do not have the ability to predict gene co-expression networks of missing cells, so the proposed algorithm provides an extra important function to make network predictions.

## 4. Real Application

### 4.1. MERFISH Mouse Hypothalamus Data

Moffitt et al. (2018) combined single-cell RNA-sequencing and a single-cell transcriptome imaging method called MERFISH to obtain expression profiles at the cellular level as well as x-y coordinates of centroid positions for cells in the mouse hypothalamic preoptic region. In the MERFISH mouse hypothalamus data, class information of cells are also available. The single-cell spatial expression data can be downloaded from https://datadryad.org/stash/dataset/doi:10.5061/dryad.8t8s248.

**Figure 5:**
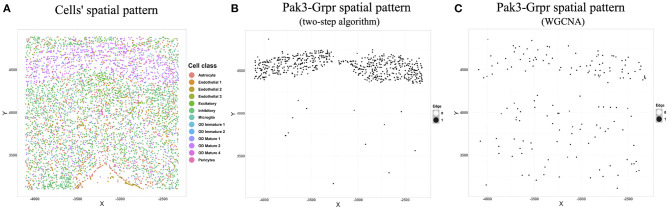
**(A)** Cells' spatial distribution pattern, where different colors of points correspond to different cell classes. **(B)** The spatial pattern of the Pak3-Crpr connection obtained by two-step algorithm. The black point reflects that an edge exists between “Pak3” and “Crpr” in that cell. **(C)** The spatial pattern of the Pak3-Crpr connection obtained by WGCNA.

**Figure 6:**
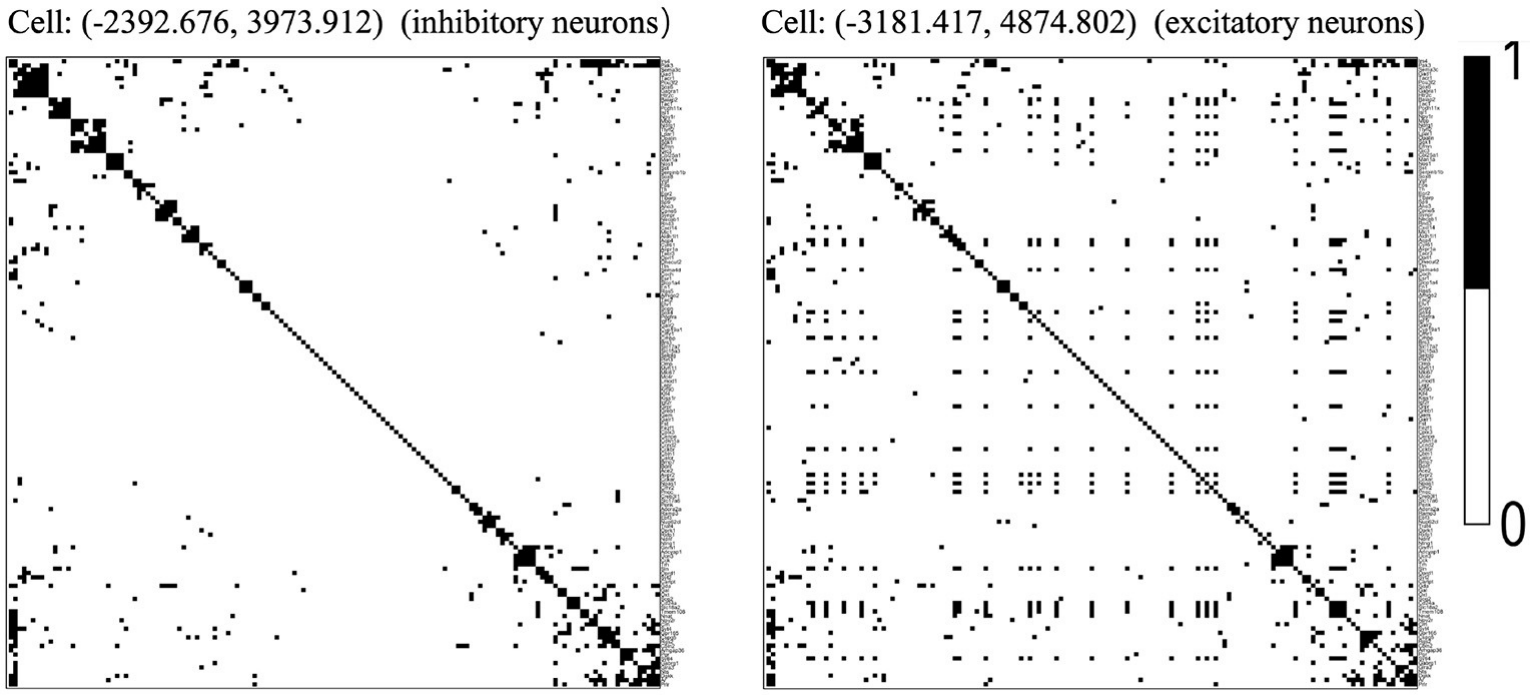
Estimated gene co-expression networks of two selected cells in the MERFISH mouse hypothalamus data: the left panel corresponds to an inhibitory cell, while the right panel corresponds to an excitatory cell.

We chose the expression data with animal id 35 and location 0.26 of the slice in bregma coordinates and removed cells labeled “Ambiguous” as well as cell types that contain less than 10 cells, resulting in 13 cell classes. The spatial pattern of the selected cells was displayed in Figure 5A. We further removed “blank” genes and genes whose expressions are zero across all the cells in one cell type, resulting in *G* = 147 genes and *n* = 4, 682 cells. Subsequently, we applied the proposed two-step algorithm with informative neighboring cell number *m*_*info*_ = 70 and threshold parameter *d* = 0.1. We randomly selected two cells from cell classes “inhibitory neurons” and “excitatory neurons,” respectively, and the gene co-expression networks of the two cells were shown in Figure 6. It is observed that the two gene co-expression networks have similar functional gene modules on the diagonal possibly because both of them are neurons. Moreover, the network of the cell in excitatory neurons is denser than the network in inhibitory neurons, and the reason may be that the gene activity in cells controlling excitement is more active than that in cells controlling inhibition.

Cell-specific gene co-expression networks can provide insightful information about how genes' degrees vary in each cell type. To show that, in excitatory neuron cells, we selected 15 genes with the most variable degrees: Sln, Baiap2, Tmem108, Oprk1, Slc17a6, Nos1, Htr2c, Irs4, Gpr165, Slc18a2, Vgf, Pgr, Ar, Gabrg1, and Gabra1. To validate the functions of the gene set, we conducted gene set enrichment analysis (Subramanian et al., 2005) based on the gene ontology (GO) database (Gene Ontology Consortium, 2004). We found several significant annotations related to the excitatory neurons including GO_MODULATION_OF_EXCITATORY_POSTSYNAPTIC_POTENTIAL (biological process), GO_EXCITATORY_SYNAPSE (cellular component), and GO_NEURON_PROJECTION (cellular component). In terms of the inhibitory neurons, we identified 15 genes with the most variable degrees: Baiap2, Sox6, Irs4, Ar, Gda, Oprk1, Isl1, Cyr61, Prlr, Glra3, Gabra1, Dgkk, Tmem108, Sln, and Ano3. Using GO annotations, the gene set is associated with inhibitory neurons-related activities including GO_INHIBITORY_EXTRACELLULAR_LIGAND_GATED_ION_CHANNEL_ACTIVITY (molecular function) and GO_NEURON_PROJECTION (cellular component). These observations show that estimated cell-specific networks have the potential to find genes with variable degrees for each cell type, which cannot be accomplished by cell-type-specific approaches.

We next illustrated the spatial feature of estimated gene co-expression networks in terms of gene-gene connections. We calculated the median degree for each gene. Gene Pak3 with the maximum median degree (31) and gene Grpr with the minimum median degree (0) were chosen for demonstration. Figure 5B shows that the Pak3-Grpr connection mainly appears in the region where “mature oligodendrocytes” are enriched. The observation indicates that the two genes may tend to work together in the mature oligodendrocytes. Actually, mutations on gene Pak3 are related to intellectual disability diseases, and its expression decreases in mature oligodendrocytes and may regulate oligodendrocyte precursor cell differentiation, as reported in a previous study (Renkilaraj et al., 2017). To demonstrate the advantage of estimating cell-specific networks, we further applied WGCNA (Zhang and Horvath, 2005) with truncation level 0.1 to obtain cell-type-specific networks. However, Figure 5C indicates that the cell-type-specific estimations by WGCNA cannot reveal the pattern provided by cell-specific estimations.

**Figure 7:**
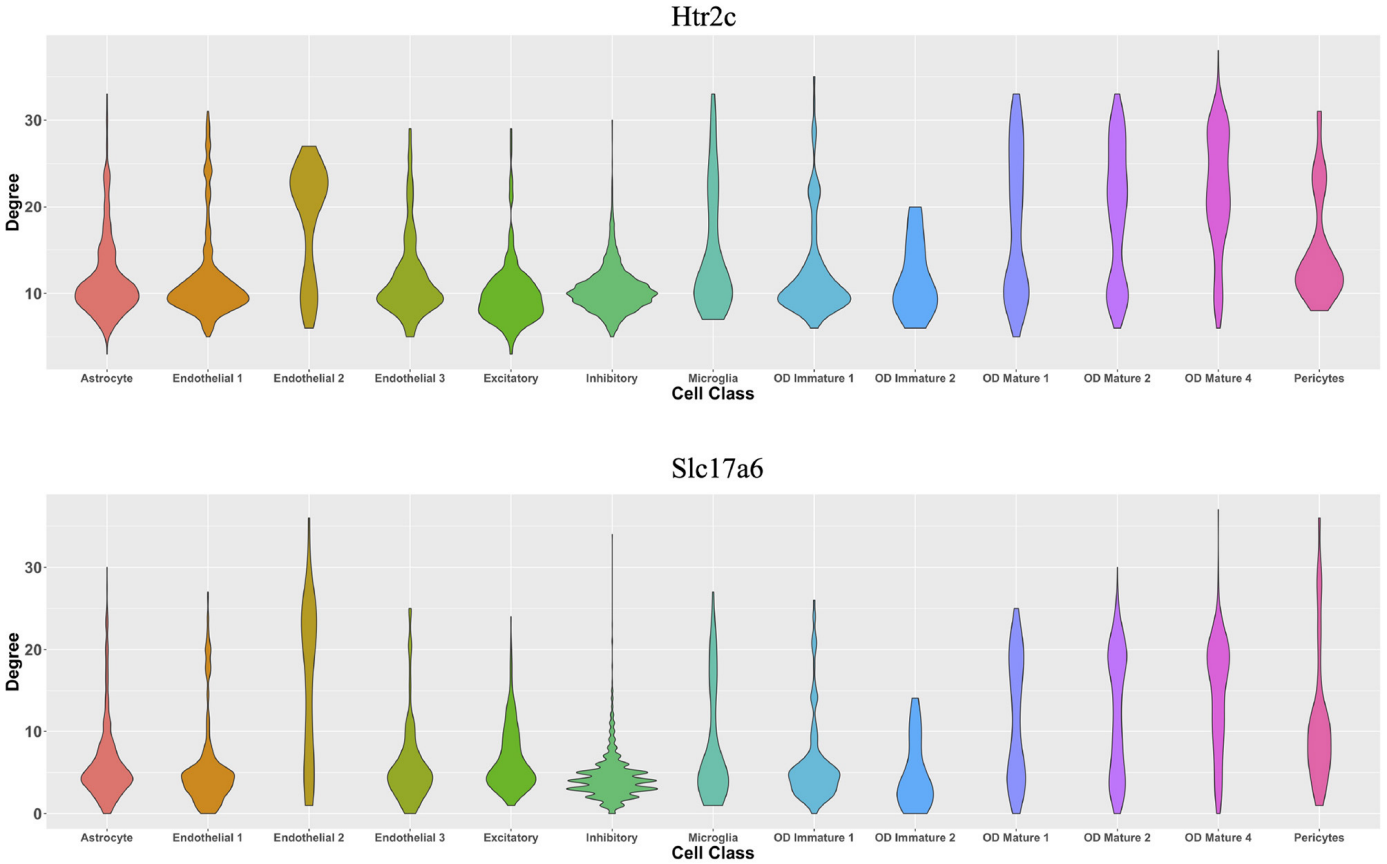
Violin plots of two genes' degree distributions across thirteen cell classes in the MERFISH mouse hypothalamus data.

From the perspective of cell types, Figure 7 demonstrates the cell-type-specific degree distributions of two genes, Htr2c and Slc17a6 (Campbell et al., 2017; Chen et al., 2017), which have the most degree variances across cells. It is observed that the degree distribution of one gene varies across cell types, and this cannot be observed by traditional cell-type-specific gene co-expression networks.

### 4.2. MERFISH U-2 OS Data

**Figure 8:**
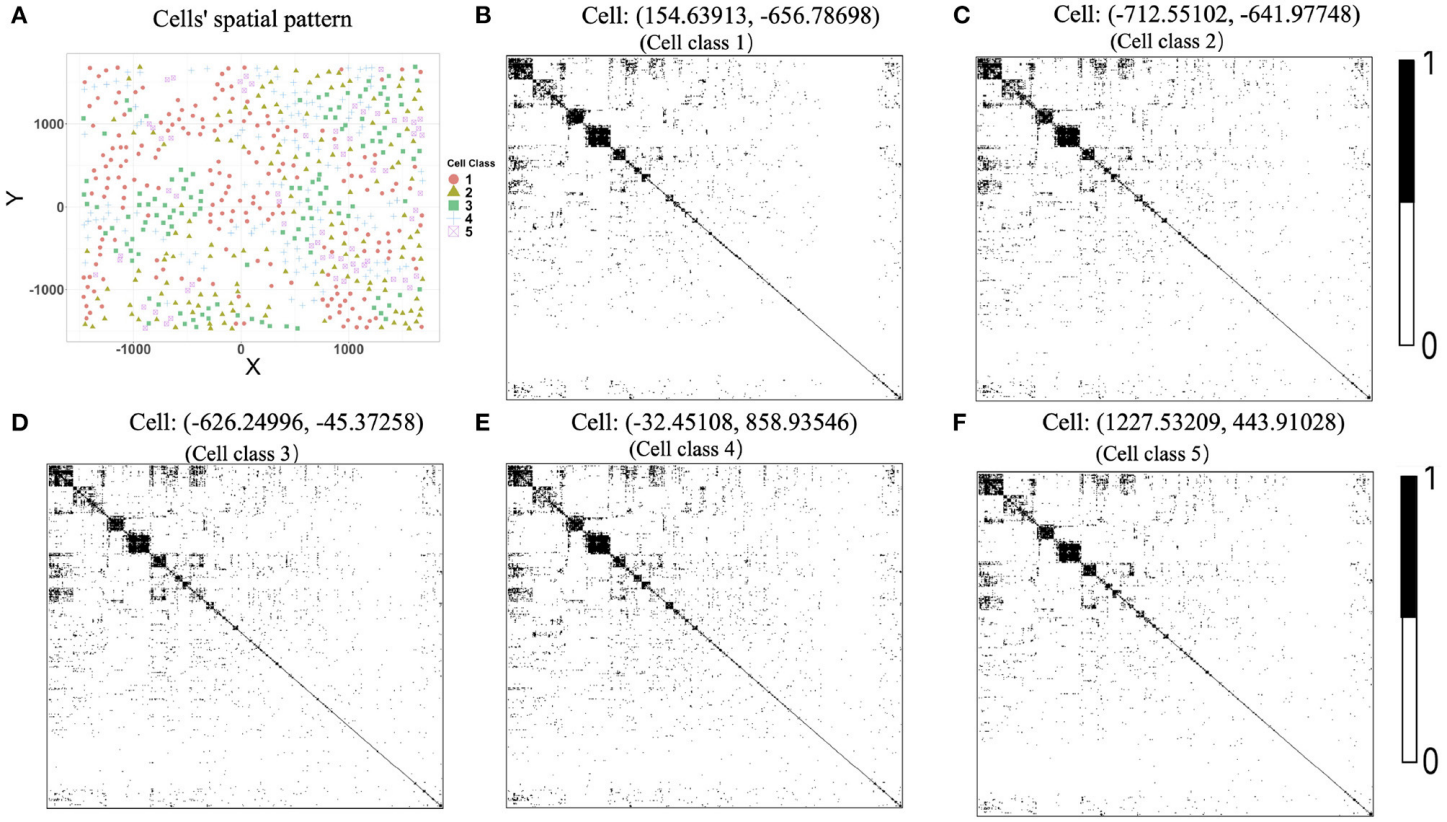
**(A)** Cells' spatial distribution pattern, where different colors and shapes of points correspond to different cell classes. **(B–F)** Estimated gene co-expression networks of five randomly selected cells in the MERFISH U-2 OS cell line data.

We further provided some simple results of the proposed algorithms on another single-cell spatial expression dataset. Xia et al. (2019) carried out the MERFISH experiments on human osteosarcoma (U-2 OS) cells, and we downloaded the expression count data from https://www.pnas.org/content/116/39/19490/tab-figures-data. The data contain expression profiles for 10,050 genes and 1,368 cells in three batches. To avoid possible influences caused by batch effects, our analysis focuses on the batch one. We first removed “blank” genes, resulting in *n* = 645 cells and *G* = 10, 050 genes. Since there is no cell-type annotation information, we first performed cell clustering procedure using Seurat (Butler et al., 2018; Stuart et al., 2019). By setting the resolution at 0.8 in Seurat clustering procedure, we obtained *K* = 5 cell classes, which is consistent with the cell type number in Xia et al. (2019). Figure 8A shows the cells' spatial distribution.

**Figure 9:**
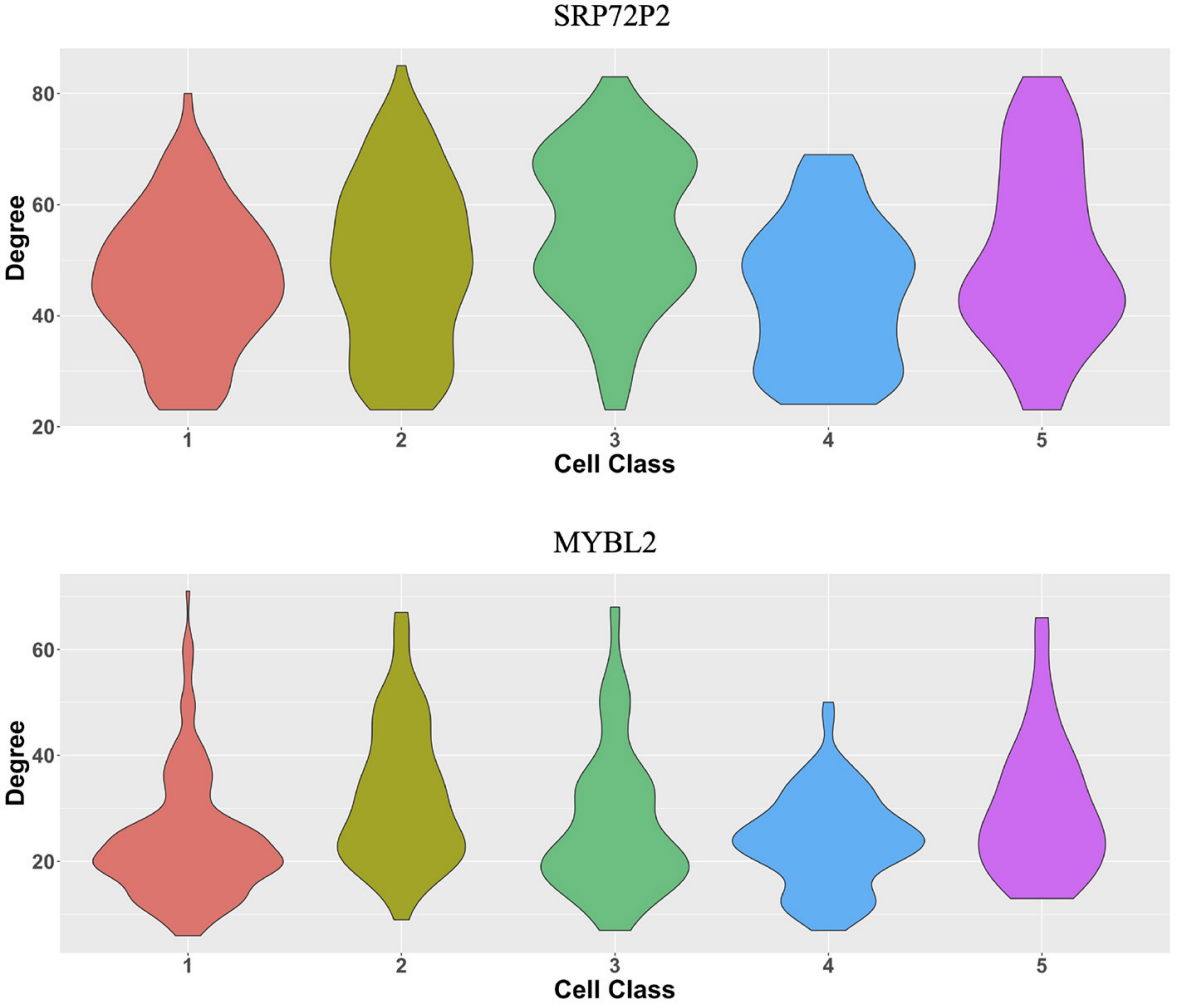
Violin plots of two genes' degree distributions across five cell classes in the MERFISH U-2 OS cell line data.

The original expression data were count data, so we normalized the data following the formula $x_{gi}\leftarrow \frac{10^{6}}{\underset{g}{\sum }x_{gi}}x_{gi}$, where *x*_*gi*_ is the expression level of gene *g* in cell *i* and then selected the most variable 500 genes to perform the proposed two-step algorithm. The informative neighboring cell number *m*_*info*_ was set to 70, and the threshold parameter *d* was set to 0.3. Accordingly, we randomly selected five cells from the five cell classes, respectively, and the gene co-expression networks of the five chosen cells were shown in Figures 8B–F. It is observed that the five gene networks from different cell types have similar gene modules. Moreover, we showed the degree distributions across five cell types for two genes, SRP72P2 and MYBL2, which have the most degree variances across cells. Figure 9 tells us that the degree distributions of the two genes not only have variation within one cell type but also change from one cell type to another.

## 5. Discussion

Recent technology advances enable us to gain deep insights into spatial cell-specific gene expressions. In this paper, we developed a simple and computationally efficient two-step algorithm to recover spatially-varying cell-specific gene co-expression networks. The simulation study shows that the proposed algorithm outperforms the traditional cell-type-specific gene network approach and cell-specific gene network estimation methods that do not employ spatial information. The application to the MERFISH data provides some interesting biological findings. In the meanwhile, there are some limitations in the proposed algorithm we aim to improve in the future work. For example, we choose a hard threshold to identify a gene-gene connection, but an adaptive threshold selection needs to be derived.

We also acknowledge that using normal distributions to fit normalized gene expression data can lose power and be suboptimal compared to directly modeling the sequencing count data via Poisson distributions (Sun et al., 2017). Fortunately, in several previous bioinformatics works, using continuous multivariate normal distributions to model normalized single-cell sequencing data (Pierson and Yau, 2015; Chen and Zhou, 2017; Wang et al., 2020) or spatial single-cell expression data (Li D. et al., 2020) can still provide key biological findings. Moreover, in terms of computation, multivariate Poisson distributions (Karlis, 2003) largely increase the computational burden. Statistically, the covariance matrix in the multivariate Poisson distribution does not have a standard conjugate prior, thus failing to obtain an analytical form of the posterior mean. In real data, the cell number is often large (~4,000 in our real application), which actually guarantees a satisfying normal approximation. Considering these issues, we chose the multivariate normal as the data distribution, but it is very interesting and challenging to extend the algorithm to directly model raw count data and we leave it for future work.

## Data Availability Statement

Publicly available datasets were analyzed in this study. MERFISH mouse hypothalamus data can be downloaded from https://datadryad.org/stash/dataset/doi:10.5061/dryad.8t8s248, and MERFISH U-2 OS cell line data is available via the link https://www.pnas.org/content/116/39/19490/tab-figures-data.

## Code Availability Statement

The codes that can reproduce results in simulation and real application are available on GitHub, https://github.com/jingeyu/CSSN_data_code. The associated CSSN package is available on GitHub, https://github.com/jingeyu/CSSN.

## Author Contributions

XL conceived the study. JY and XL developed the method, analyzed the real data, and wrote the paper. JY implemented the algorithm, prepared the software, and conducted simulation. Both authors contributed to the article and approved the submitted version.

## Conflict of Interest

The authors declare that the research was conducted in the absence of any commercial or financial relationships that could be construed as a potential conflict of interest.
